# Epigenetic and antitumor effects of platinum(IV)-octanoato conjugates

**DOI:** 10.1038/s41598-017-03864-w

**Published:** 2017-06-16

**Authors:** Vojtech Novohradsky, Ilaria Zanellato, Cristina Marzano, Jitka Pracharova, Jana Kasparkova, Dan Gibson, Valentina Gandin, Domenico Osella, Viktor Brabec

**Affiliations:** 1 Institute of Biophysics, Academy of Sciences of the Czech Republic, v.v.i., Kralovopolska 135, CZ-61265 Brno, Czech Republic; 2 Dipartimento di Scienze e Innovazione Tecnologica, Universita del Piemonte Orientale, “A. Avogadro”Viale T. Michel 11, 15121 Alessandria, Italy; 3 Dipartimento di Scienze del Farmaco, Universita di Padova, Via Marzolo 5, 35131 Padova, Italy; 4 Department of Biophysics, Centre of the Region Hana for Biotechnological Agricultural Research, Faculty of Science, Palacky University, 17. listopadu 12, CZ-77146 Olomouc, Czech Republic; 5 Institute for Drug Research, School of Pharmacy, The Hebrew University, Jerusalem, 91120 Israel

## Abstract

We present the anticancer properties of *cis*, *cis*, *trans*-[Pt(IV)(NH_3_)_2_Cl_2_(OA)_2_] [Pt(IV)diOA] (OA = octanoato), Pt(IV) derivative of cisplatin containing two OA units appended to the axial positions of a six-coordinate Pt(IV) center. Our results demonstrate that Pt(IV)diOA is a potent cytotoxic agent against many cancer cell lines (the IC_50_ values are approximately two orders of magnitude lower than those of clinically used cisplatin or Pt(IV) derivatives with biologically inactive axial ligands). Importantly, Pt(IV)diOA overcomes resistance to cisplatin, is significantly more potent than its branched Pt(IV) valproato isomer and exhibits promising *in vivo* antitumor activity. The potency of Pt(IV)diOA is a consequence of several factors including enhanced cellular accumulation correlating with enhanced DNA platination and cytotoxicity. Pt(IV)diOA induces DNA hypermethylation and reduces mitochondrial membrane potential in cancer cells at levels markedly lower than the IC_50_ value of free OA suggesting the synergistic action of platinum and OA moieties. Collectively, the remarkable antitumor effects of Pt(IV)diOA are a consequence of the enhanced cellular uptake which makes it possible to simultaneously accumulate high levels of both cisplatin and OA in cells. The simultaneous dual action of cisplatin and OA by different mechanisms in tumor cells may result in a markedly enhanced and unique antitumor effects of Pt(IV) prodrugs.

## Introduction

Cisplatin and its homologs are powerful anticancer drugs, but their therapeutic index is very narrow due to heavy side effects. Currently, the metallodrug research is mainly focused on Pt(IV) complexes that act as prodrugs that are activated by an intracellular 2e^−^ reduction resulting in loss of the two axial ligands and generation of the corresponding cytotoxic Pt(II) species^1, 2^. As the octahedral arrangement is more kinetically inert, Pt(IV) complexes are less deactivated by platinophiles (off-target reactions) than their Pt(II) counterparts. Thus, a higher fraction of the Pt(IV) complexes may reach tumor cells intact, where they undergo reduction by intracellular reductants^3, 4^. Their axial ligands can affect the cellular accumulation of Pt(IV) compounds due to their enhanced lipophilicity (the influx occurs mainly by passive diffusion)^5^. Moreover, these axial ligands can be biologically active vectors towards tumor tissue or adjuvant (synergistic) drugs^6–8^.

Early *in cellulo* studies showed that the cisplatin-based Pt(IV) complexes, having medium-chain fatty acids (MCFAs) axial ligands, namely octanoato (OA) and its branched isomer valproato (VPA), possess remarkable antiproliferative activity^9, 10^. It has been shown^11, 12^ that the enhanced cytotoxicity of the *cis*, *cis*, *trans*-[Pt(IV)(NH_3_)_2_Cl_2_(VPA)_2_] conjugate [Pt(IV)diVPA] is a consequence of several processes. They involve enhanced cellular accumulation, downregulation of histone deacetylases (HDACs) and yet other biochemical processes (not involving HDACs) which may potentiate antitumor effects. On the other hand, the higher activity of *cis*, *cis*, *trans*-[Pt(IV)(NH_3_)_2_Cl_2_(OA)_2_] complex [Pt(IV)diOA], compared with Pt(IV)diVPA, was so far attributed only to the higher lipophilicity of the former and consequently to enhanced cellular accumulation^10^ that occurs by passive diffusion^13^. No other biochemical processes triggered by OA released from Pt(IV)diOA by its intracellular reduction contributing to its antitumor effects have been identified. Thus, the question of why Pt(IV)diOA is more potent than its Pt(IV)diVPA (with bioactive ligands) remains unresolved and deserves deeper biochemical investigations.

OA, also known as caprylic acid, has antibacterial activity^14^. Moreover, OA was recently reported to act as an HDAC inhibitor like VPA, albeit less effective^15^. Additionally, the prediction of *in vivo* anticancer activity of such lipophilic Pt(IV) complexes by *in vitro* cell culture experiments is not trivial taking into consideration the fact that these prodrugs were designed for oral administration via absorption through the gastrointestinal tract^16^.

**Figure 1:**
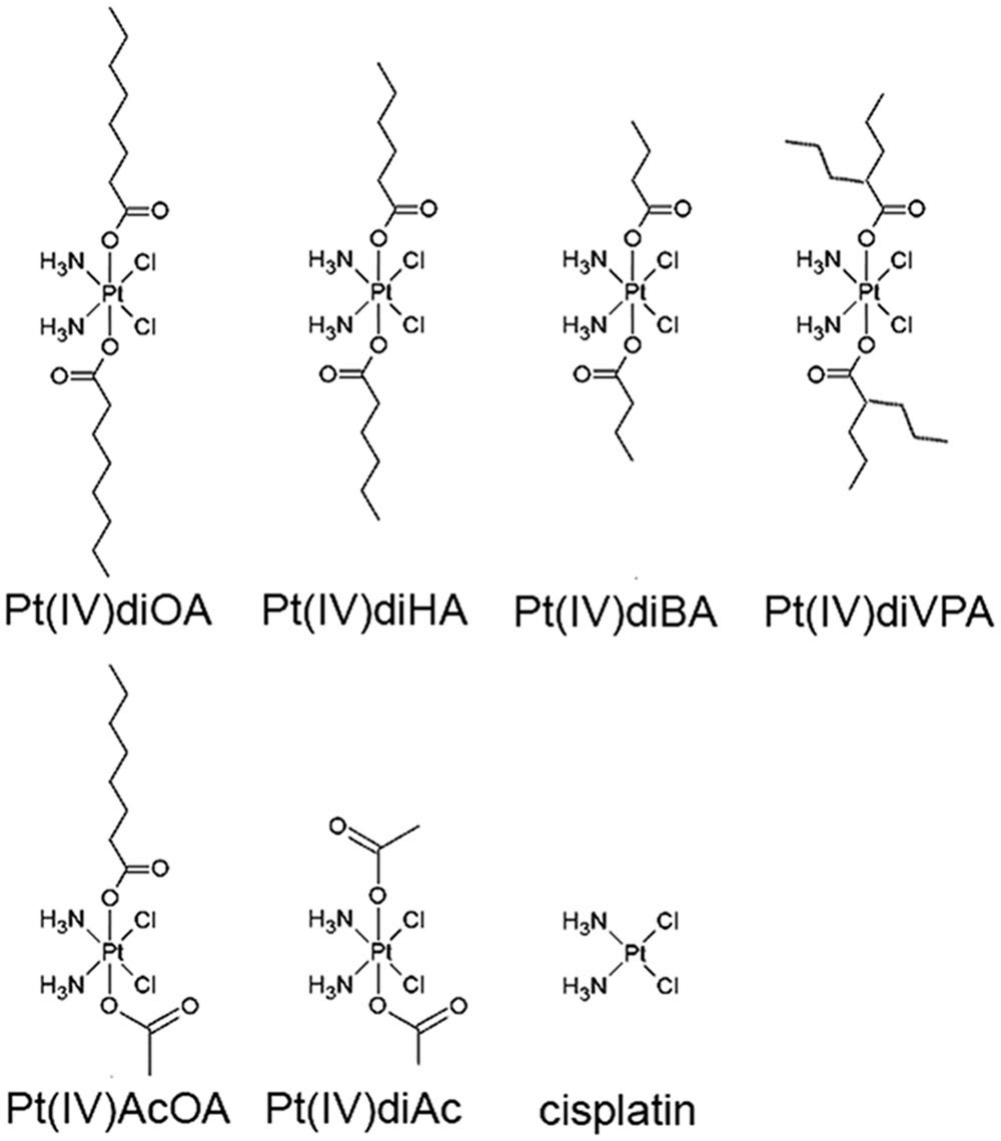
Schematic representation of the platinum compounds used in the present work.

Here we report that the markedly enhanced toxic effects of Pt(IV)diOA in tumor cells are connected not only with enhanced accumulation of this prodrug in tumor cells, but notably also with epigenetic changes. These mechanistic studies were extended by evaluations of the *in vivo* efficacy of a series of Pt(IV) derivatives of cisplatin. These derivatives contained carboxylato axial ligands of increasing lipophilicity, namely C4, butanoato (BA); C6, hexanoato (HA); and C8 (OA and VPA) (Fig. 1) evaluated in a syngeneic murine xenograft model of lung cancer using Lewis lung carcinoma cells. Our results have broader implications for molecular pharmacology given the widespread use of platinum drugs in cancer therapy. Our studies suggest that strategies based on platinum(IV) prodrugs combined with epigenetically active compounds synergistically improve efficacy.

## Results

### Biochemical mechanisms in tumor cells treated with Pt(IV)diOA which potentiate its cytotoxic activity

#### Cytotoxicity

Cytotoxic activity of the compounds tested in the present work was screened on the panel of malignant and nonmalignant cell lines using the colorimetric MTT assay and 72 h of drug incubation period. Notably, significant differences in the killing ability of the compounds were observed (Table 1). After the treatment of the cells with free octanoic acid, the IC_50_ values (concentration of compound that causes death in 50% of cells) ranged from 0.71 mM in human prostate adenocarcinoma cells LNCaP up to 16 mM in breast adenocarcinoma cells MCF7. In contrast, the IC_50_ values found for Pt(IV)diOA were in the nanomolar range in accord with previously published data^10^. The IC_50_ values found for Pt(IV)diOA conjugate ranged from 17 nM in colon carcinoma cells HCT-116 up to 140 nM in nonmalignant Chinese hamster ovary cells (an epithelial cell line) CHO-K1, i.e. that these IC_50_ values were by one or two orders of magnitude lower than those found for cisplatin. Similarly, conjugating Pt(IV) derivative of cisplatin with one OA and one Ac in the axial positions led to a significant improvement of cytotoxicity compared with cisplatin. The IC_50_ values found for Pt(IV)AcOA were in the submicromolar range except for the IC_50_ value found for CHO-K1 cells (IC_50_ was ~1 µM). The IC_50_ values for Pt(IV)diOA were significantly lower than those of its isomer Pt(IV)diVPA in the A2780 cell line (0.041 ± 0.001 vs. 0.25 ± 0.09), A2780cisR (0.029 ± 0.002 vs. 0.3 ± 0.1) and MCF-7 (0.038 ± 0.006 vs. 0.9 ± 0.3)^11^.

**Table 1:** Cytotoxicity. IC_50_ mean values obtained for platinum complexes (μM) and octanoic acid (mM)^a^.

	A2780	A2780cisR	RF^b^	CHO-K1	MCF-7	LNCaP
Pt(IV)diOA	0.041 ± 0.001	0.029 ± 0.002	0.7	0.14 ± 0.02	0.038 ± 0.006	0.052 ± 0.005
Pt(IV)AcOA	0.35 ± 0.05	0.37 ± 0.06	1.1	1.1 ± 0.2	0.50 ± 0.04	0.27 ± 0.10
Pt(IV)diAc	5.3 ± 1.0	22.0 ± 3.1	4.2	14.5 ± 0.7	51.0 ± 4.0	≤50
cisplatin	2.8 ± 0.1	13.8 ± 0.1	4.9	19.9 ± 1.0	17.4 ± 1.9	8.54 ± 1.78
OA (mM)	1.46 ± 0.10	0.76 ± 0.02	0.5	5.9 ± 0.7	16.23 ± 0.56	0.71 ± 0.12
	**PNT1a**	**HSF**	**MRC-5** (**PD30**)		**HCT-116**	
Pt(IV)diOA	0.022 ± 0.001	0.077 ± 0.001	0.052 ± 0.003		0.017 ± 0.003	
Pt(IV)AcOA	0.150 ± 0.003	0.957 ± 0.019	0.409 ± 0.215		0.93 ± 0.02	
Pt(IV)diAc	2.48 ± 0.10	≤50	≤50		35.3 ± 1.4	
cisplatin	2.50 ± 0.32	68.98 ± 0.87	10.60 ± 0.38		5.9 ± 0.9	
OA (mM)	2.07 ± 0.03	9.74 ± 0.03	7.67 ± 0.61		3.84 ± 0.39	
Decitabine					7.5 ± 0.3	

^a^Cytotoxic effects of tested compounds were determined by MTT assay. The drug-treatment period was 72 h. The results are expressed as mean values ± SD from three independent experiments, each performed in triplicate.

^b^RF – Resistance factor, defined as IC_50_ (resistant, A2780cisR)/IC_50_ (sensitive, A2780).

Interestingly, the lowest resistance factor (RF) was found for free octanoic acid followed by the Pt(IV) conjugate Pt(IV)diOA. This observation suggests that the OA ligands in Pt(IV) prodrugs help overcome acquired resistance of tumor cells to cisplatin.

#### Cellular accumulation

The cellular accumulation of platinum from Pt(IV) derivatives of cisplatin in human ovarian carcinoma A2780 and human colon carcinoma HCT-116 cell lines was studied to investigate a possible relationship between the cellular uptake and *in vitro* cytotoxicity of the complexes. Cellular concentrations of platinum were determined by inductively coupled plasma mass spectrometry (ICP-MS) after 24 h of exposure to the complexes at the concentration of 0.35 µM. This concentration was chosen as a compromise between limit of quantitation (LOQ) of platinum in our biological samples by ICP-MS and the high toxicity of Pt(IV)diOA which could misrepresent the results. The results are summarized in Table 2.

**Table 2:** Cellular platinum accumulation/distribution in A2780 and HCT-116 cells and log P (octanol/water) values obtained by shake flask method.

A2780	Total ng Pt/10^6^ cells^a,e^	Cytosol pg Pt/µg protein^b,e^	Nucleus pg Pt/µg protein^c,e^	log P^d,e^
Pt(IV)diOA	12.9 ± 0.3	138 ± 12	173 ± 15	0.92 ± 0.03
Pt(IV)AcOA	4.0 ± 0.9	28 ± 4	27 ± 4	0.59 ± 0.04
Pt(IV)diAc	0.45 ± 0.09	3.0 ± 0.4	3.0 ± 0.5	−1.85 ± 0.09
cisplatin	0.8 ± 0.2	5.5 ± 0.9	3.0 ± 0.7	−2.25 ± 0.04
**HCT-116**				
Pt(IV)diOA	29.2 ± 0.3	59 ± 7	189 ± 9	
Pt(IV)AcOA	6.0 ± 0.9	48 ± 2	55 ± 7	
Pt(IV)diAc	0.19 ± 0.04	2.1 ± 0.3	4.6 ± 0.5	
cisplatin	0.9 ± 0.1	3.6 ± 0.2	4.1 ± 0.3	

^a^The table shows the cellular platinum accumulation into A2780 and HCT-116 cells after 24 h of exposure to 0.35 µM of the compounds.

^b,c^Platinum accumulation in different pools of A2780 and HCT-116 cells (nucleus^b^ and cytosol^c^).

^d^log P values obtained by shake flask method.

^e^Results are expressed as the mean ± SD for three independent experiments with triplicate for each run.

The lowest level of platinum associated with the cells was observed after exposure to Pt(IV)diAc followed by cisplatin. The replacement of axial acetato groups in Pt(IV)diAc by OA resulted in a significant enhancement of the level of platinum associated with the cells. This enhancement was most pronounced for Pt(IV)diOA. To quantify the hydrophobicity of the platinum complexes, the shake-flask method was used to measure partition coefficients (log P) values for octanol/water. The log P values for cisplatin Pt(IV)diAc, Pt(IV)AcOA, and Pt(IV)diOA are shown in Table 2. Cisplatin and Pt(IV)diAc are hydrophilic (partition preferentially into water). In contrast, the replacement of axial acetato ligands in Pt(IV)diAc by one or two OA groups transformed this Pt(IV) derivative of cisplatin into hydrophobic species Pt(IV)AcOA and Pt(IV)diOA (partition preferentially into octanol). Interestingly, logP values found for Pt(IV)AcVPA and Pt(IV)diVPA^11^ were 0.17 and 0.25 indicating that Pt(IV) complexes containing branched C8 chain were less hydrophobic than their octanoato derivatives containing linear C8 aliphatic chain. This is also evident by noting that despite having six carbons less, Pt(IV)AcOA has similar log P value as Pt(IV)diVPA. The latter result is consistent with the finding that logP (estimated using ChemDraw Ultra v8.0 software) for free OA is higher than that of free VPA.

Cellular uptake studies were also carried out to measure the accumulation of platinum species in cytosol and nucleus. A2780 and HCT-116 cells were treated with 0.35 µM cisplatin and its Pt(IV) derivatives for 24 h, and the cells were fractionated into cytosol and nucleus. The platinum levels in each pool were measured by ICP-MS. The results are presented in Table 3. The extent of accumulation of both OA conjugated Pt(IV) prodrugs into the cytosol and nucleus fractions was markedly higher than that of cisplatin or Pt(IV)diAc.

**Table 3:** Platinum content on DNA isolated from A2780 and HCT-116 cells treated with cisplatin and its Pt(IV) derivatives^a^.

A2780	pg Pt/µg DNA^b^	% from the total intracellular Pt amount^c^
Pt(IV)diOA	43.3 ± 5.3	13.0
Pt(IV)AcOA	8.0 ± 1.4	5.0
Pt(IV)diAc	1.1 ± 0.4	1.9
cisplatin	1.5 ± 0.6	2.0
**HCT-116**		
Pt(IV)diOA	123.1 ± 5.1	11.9
Pt(IV)AcOA	19.8 ± 3.6	4.8
Pt(IV)diAc	1.3 ± 0.1	2.0
cisplatin	1.6 ± 0.2	2.2

^a^Cells were treated for 24 h with tested compounds (0.35 µM).

^b^Platinum content on DNA from A2780 and HCT-116 expressed in pg of Pt per µg of DNA. Results are expressed as the mean ± SD for three independent experiments with triplicate for each run.

^c^The values represent platinum content on DNA from A2780 and HCT-116 cells expressed as the percentage of the total platinum accumulated inside these cells.

The results of cellular accumulation of platinum from Pt(IV) prodrugs and cisplatin are consistent with the premise that hydrophobic rather than hydrophilic platinum compounds penetrate more easily the cytoplasmic membrane, which leads to their increased cellular accumulation. The presence of Ac ligands in axial positions of Pt(IV) derivative of cisplatin led to only a slight decrease of hydrophilicity, but Pt(IV)diAc was still hydrophilic. On the other hand, the replacement of axial acetato ligands in Pt(IV)diAc by one or two OA groups transformed this Pt(IV) complex into lipophilic compounds Pt(IV)AcOA and Pt(IV)diOA.

#### DNA-bound platinum in cells exposed to Pt(IV) derivatives of cisplatin containing axial Ac and OA ligands

As for antitumor cisplatin and its direct derivatives, DNA is believed to be the target also for Pt(IV) prodrugs examined in the present work. For this reason, DNA from A2780 and HCT-116 cells, after the exposure to 0.35 µM cisplatin and its Pt(IV) analogs for 24 h, was isolated and the levels of platinum on DNA were determined by ICP-MS (Table 3). The platinum content of DNA from the A2780 cells treated with Pt(IV)AcOA and Pt(IV)diOA was approximately 7.3 and 39.4-fold greater, respectively than that of the cells treated with Pt(IV)diAc; or approximately 5.3- and 28.9-fold greater, respectively, than that of the cells treated with cisplatin. Similarly, the platinum content of DNA from the HCT-116 cells treated with Pt(IV)AcOA and Pt(IV)diOA was approximately 15.2 and 94.7-fold greater, respectively than that of the cells treated with Pt(IV)diAc; or approximately 12.4- and 76.9-fold greater, respectively, than that of the cells treated with cisplatin.

We made a rough estimate of the percentage of the amount of platinum in the DNA fraction of the total amount of intracellular platinum considering data in Tables 2 and 3. Of the total intracellular Pt, a higher fraction of platinum from Pt(IV)AcOA or Pt(IV)diOA was bound to DNA in comparison with the treatment with Pt(IV)diAc or cisplatin (Table 3). In aggregate, the presence OA axial ligands in the Pt(IV) derivatives of cisplatin markedly increased targeting DNA in tumor cells, which supports the view that DNA is the pharmacological target also for the class of Pt(IV) derivatives of cisplatin containing OA axial ligands.

#### Inhibition of histone deacetylase activity

**Figure 2:**
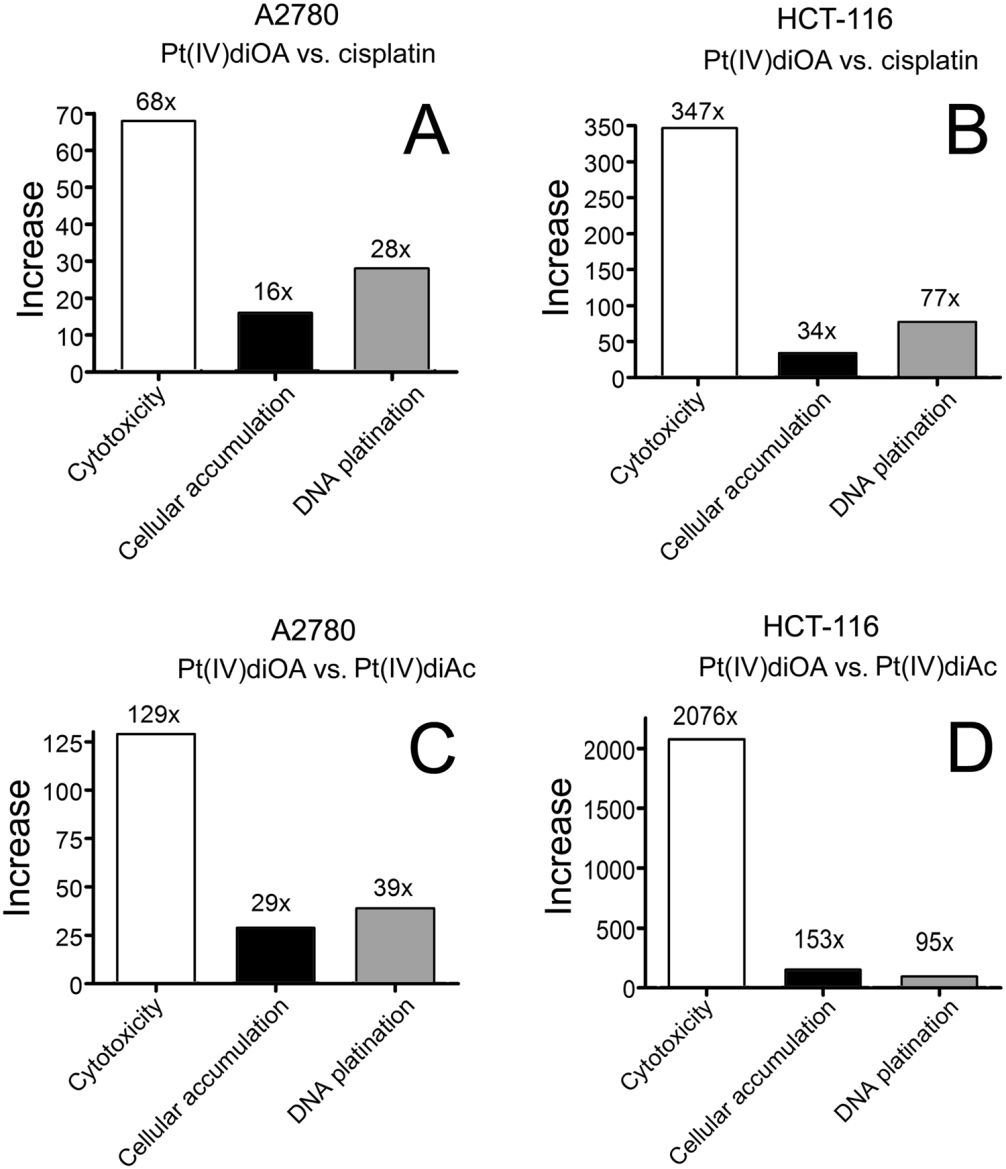
Increase of cytotoxicity, intracellular accumulation and DNA platination in A2780 (**A**,**C**) and HCT-116 (**B**,**D**) cells treated with Pt(IV)diOA in comparison with the treatment with cisplatin (**A**,**B**) or Pt(IV)diAc (**C**,**D**).

The data in Tables 1 and 2 show that presence of OA axial ligands in the Pt(IV) derivatives of cisplatin results in a substantial increase in platinum accumulation in cells in comparison with the treatment of the cells with cisplatin or its Pt(IV) derivative containing biologically inactive axial acetate ligands [Pt(IV)diAc]. However, this enhanced accumulation does not fully justify the corresponding marked enhancement of cytotoxicity (Fig. 2).

**Figure 3:**
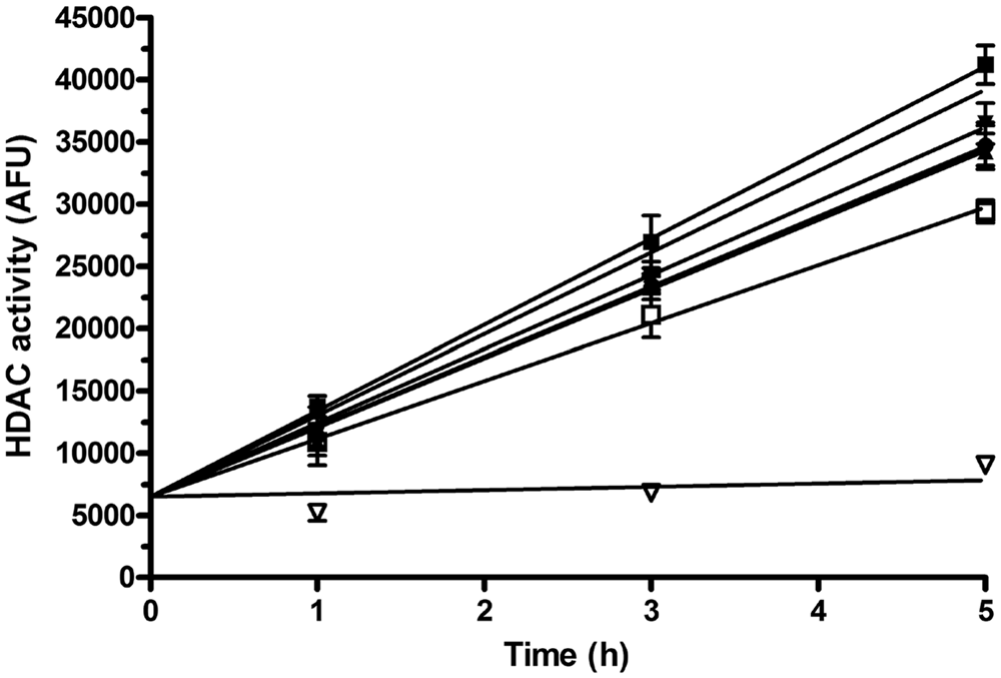
Activity of HDAC enzymes in HCT-116 cells treated with the compounds tested in the present work; their concentration was 1 µM except the concentration of free OA, which was 1 mM. Symbols: ■, control; ▲, Pt(IV)diOA; ▼, Pt(IV)AcOA; ⧫, Pt(IV)diAc; ●, cisplatin; □, OA (1 mM); ▽, trichostatin A (well known HDAC inhibitor used as the positive control). The data represent a mean from three independent experiments, each performed in quadruplicate. The error bars are the SDs.

Hence, the enhanced cytotoxicity of Pt(IV) derivatives of cisplatin containing axial OA ligands (Table 1) was not only a consequence of their enhanced accumulation in cells. In other words, yet another factor in addition to enhanced cellular accumulation had to be responsible for enhanced cytotoxicity of these Pt(IV) prodrugs compared with Pt(IV)diAc or cisplatin. It was shown^11, 12^ in the case of other Pt(IV) derivatives of cisplatin, having the branched isomer of OA, i.e. VPA, that its enhanced cytotoxicity is a consequence of several processes involving besides enhanced cellular accumulation also epigenetic processes. These epigenetic processes consist in downregulation of HDACs and yet other biochemical processes triggered by VPA released in cells by the reduction of the Pt(IV) prodrug. Moreover, it has also been shown^15^ that non-branched MCFAs may act as HDAC inhibitors. Therefore, we first examined whether the effects of Pt(IV) derivatives of cisplatin containing one or two axial OA ligands reflect their ability to inhibit HDACs in intact cells. HDAC activity was analyzed in HCT-116 cells and confirmed by an independent set of experiments on A2780 cells (data not shown) by using a commercial HDAC fluorimetric cellular activity kit as described in the section Materials and methods. Our analysis (Fig. 3) confirmed that the efficiency of the Pt(IV) derivatives (1 µM) Pt(IV)diOA, Pt(IV) AcOA and Pt(IV)diAc to inhibit HDAC activity was very small and was in this respect similar to that of cisplatin. Interestingly, the moderate inhibitory effect of free OA was observed, but only at the concentration of this agent 1000-fold higher.

#### Analysis of DNA methylation

Other well studied epigenetic mechanisms involved in biological effects of some anticancer drugs are connected with DNA methylation, a mechanism which inactivates tumor suppressor genes by methylating their promoters^17^. DNA methyltransferase (DNMT) inhibitors are even at a more clinically advanced stage of development than the inhibitors of histone deacetylases or histone methyltransferases^18^ making DNA methylation a promising target for anticancer therapies^19^.

**Figure 4:**
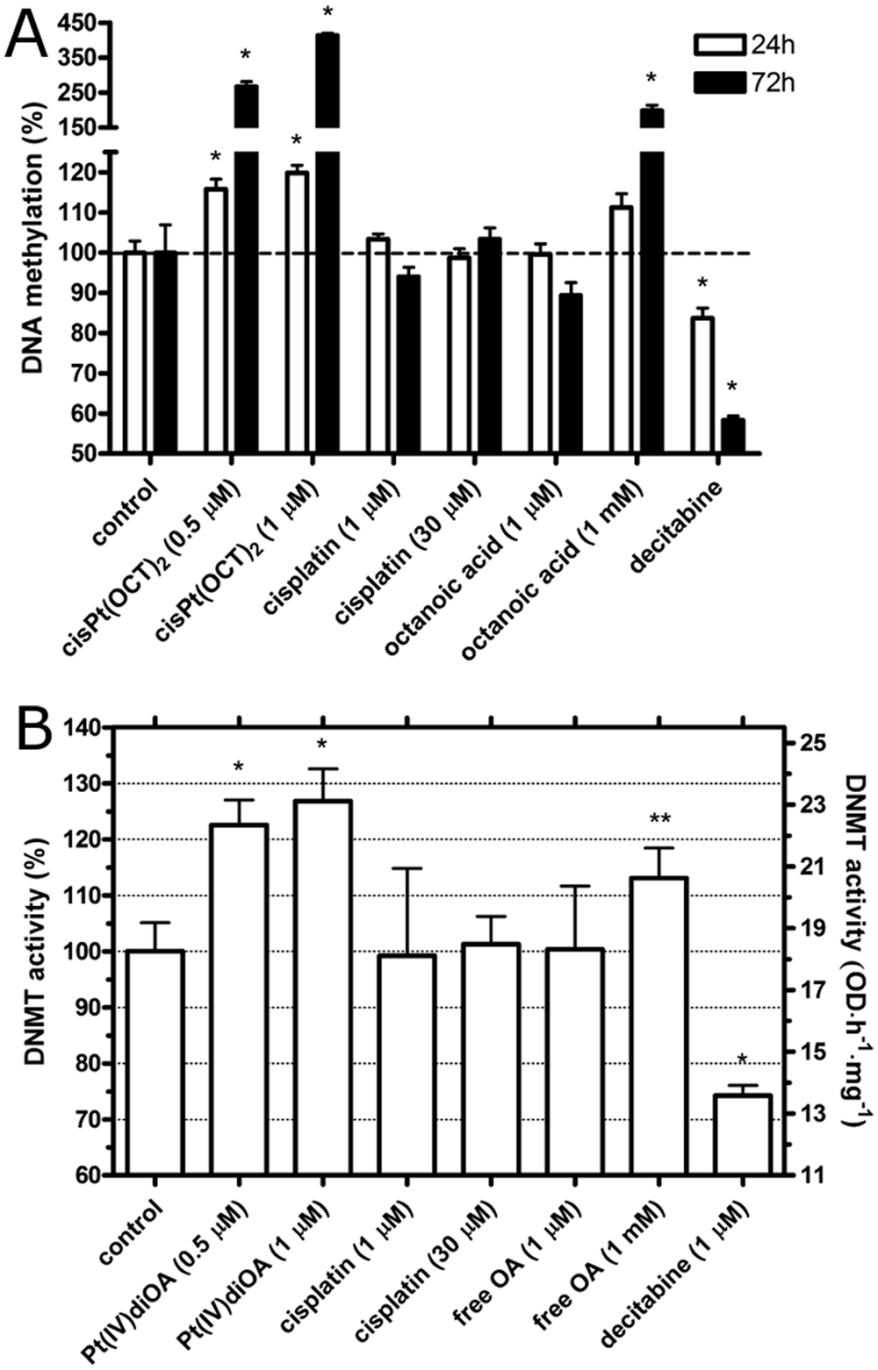
Analysis of DNA methylation. (**A**) DNA was isolated from HCT-116 cells after the exposure to the agents at the concentrations indicated in the graph for 24 or 72 h. The levels of DNA methylation in the control was taken as 100%. The bars represent mean values from three independent experiments each performed in octuplicate. The error bars are the SDs. (**B**) DNMT activity in colon carcinoma cells HCT-116 treated for 24 h with the compounds tested in the present work. The DNMT activity in the control, untreated cells was taken as 100%. Error bars are the SDs and the stars at the top of bars denote a significant difference from the control with (*p < 0.05; **p < 0.1), calculated using nonparametric student’s t-test. The bars represent mean values from three independent experiments, each performed in quadruplicate.

To find out whether Pt(IV) derivatives of cisplatin containing axial OA ligands can affect the methylation pattern of DNA in human carcinoma cells, we investigated whether treatment of HCT-116 cells with Pt(IV)diOA affects DNA methylation. Human colon adenocarcinoma cells HCT-116 were chosen as the model cellular system suitable for DNA methylation analysis because: (i) this human colorectal cancer cell line has been used as a prototypic model for analyses of the genetic basis of cancer, and the role of epigenetic changes in tumorigenesis; (ii) they have a propensity to both global DNA hypomethylation and to silence genes through promoter focal hypermethylation^20, 21^. HCT-116 cells were treated with the compounds at the concentrations indicated in Fig. 4A for 24 or 72 h and DNA was isolated for the complete methylation analysis as described in the experimental section. Decitabine (5-aza-2′-deoxycytidine), a hypomethylating agent which hypomethylates DNA by inhibiting DNMT, was used in these experiments as a negative control^22^. The IC_50_ values found for this agent in HCT-116 cells (the drug-treatment period was 72 h) was (7.5 ± 0.3) µM (Table 1).

As expected a considerably lowered level of DNA methylation was observed after the treatment with 1 µM decitabine^23, 24^. The opposite effect was observed after treatment with Pt(IV)diOA, where distinct DNA hypermethylation was observed. This effect was concentration dependent (Fig. 4A). The treatment with 0.5 or 1.0 µM Pt(IV)diOA for 24 h increased the methylation of DNA by 15 or 20%, respectively relative to the untreated control (Fig. 4B). On the other hand, the treatment with 0.5 or 1.0 µM Pt(IV)diOA for 72 h increased the methylation of DNA by 27 or 41%, respectively. A non-significant change was observed when the cells were treated with cisplatin for 72 h even at the concentration so high as 30 µM in accord with the previously published results^25^. This high concentration of cisplatin assured the intracellular concentration of platinum which was approximately identical to that when the cells were treated with one µM Pt(IV)diOA under identical experimental conditions. These control experiments support the view and are consistent with the hypothesis that DNA hypermethylation induced by 0.5 or 1.0 µM Pt(IV)diOA (Fig. 4A) was not due to the enhanced intracellular accumulation of platinum when the cells were treated with this Pt(IV) prodrug. The data obtained for free OA indicate that this MCFA if delivered into the cells in a sufficiently high concentration is also capable of inducing DNA hypermethylation.

It was shown in our previous report^11^ that the total accumulation of free VPA after 24 h exposure of the cells was more than three orders of magnitude lower than after comparable exposure to Pt(IV)diVPA since at neutral pH VPA is monoanionic and therefore does not efficiently accumulate in tumor cells. The non-branched isomer of VPA, OA, is monoanionic as well so that it is reasonable to expect that free OA will accumulate in tumor cells with markedly reduced efficiency in comparison with lipophilic Pt(IV) derivatives of cisplatin containing axial OA ligands. This markedly reduced efficiency to accumulate in tumor cells may explain the very low cytotoxicity of free OA (milimolar IC_50_ values, Table 1) and very low effect of free OA on DNA methylation in cells which is manifested only at markedly higher concentrations of this free MCFA (milimolar).

Our further experiments were aimed at finding whether Pt(IV)diOA can affect the activity of DNMT enzymes in HCT-116 cells treated with this prodrug. In these experiments, the total activity of DNMT enzymes was determined in nuclear extracts prepared from HCT-116 cells which were treated with the platinum compounds, free OA or decitabine for 24 h (Fig. 4B).

The enhanced activity of DNMT enzymes was observed when the cells were treated with 0.5 or 1.0 µM Pt(IV)diOA or free 1 mM OA (Fig. 4B). On the other hand, treatment of HCT-116 cells with 1.0 µM Pt(IV) derivative of cisplatin containing ineffective acetato axial ligands or 1.0 or 30 µM cisplatin had no significant effect on the activity of DNMT enzymes. These results show that Pt(IV)diOA efficiently potentiates DNMT activity resulting in global DNA hypermethylation (Fig. 4B).

#### Analysis of mitochondrial membrane potential

**Figure 5:**
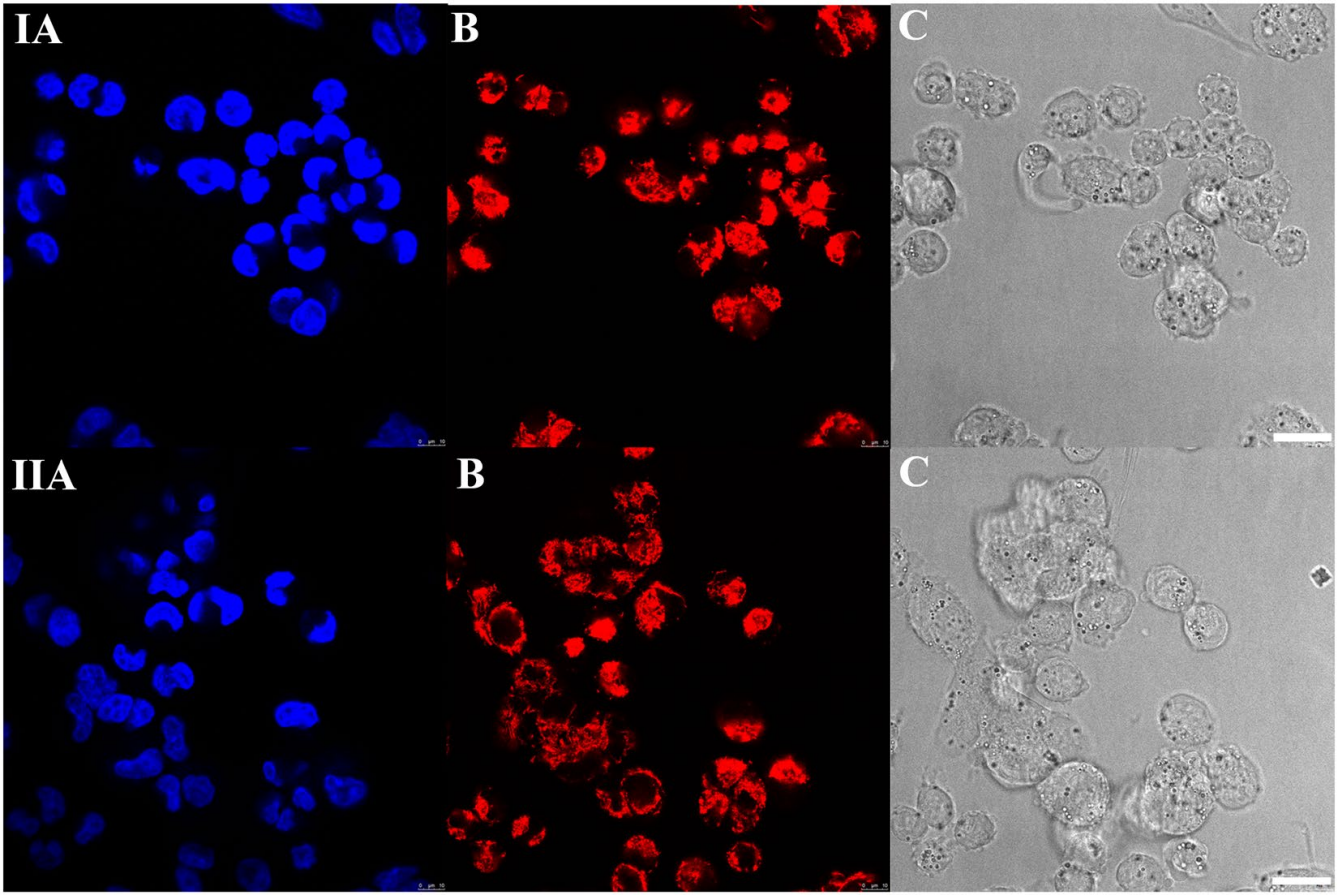
Confocal microphotographs of HCT-116 cells with nucleus stained with Hoechst 33342 (**A**) and mitochondria stained with TMRE (**B**). Cells were treated for 5 h with 1 µM of Pt(IV)diOA (upper row I). Control, untreated cells are shown in the row II (bottom) of the figure. Cells stained with Hoechst 33342 or TMRE were visualized in the bright field mode (**C**). Scale bars represent 20 µm. Figures are the representatives of at least 10 individual scans of different sample areas. Three independent experiments were performed in triplicate.

It has been shown that several cytotoxic Pt(IV) derivatives of cisplatin or oxaliplatin affect mitochondrial functions and the mitochondrial membrane potential Δ*ψ*_m_^26–28^. The loss of Δ*ψ*_m_ represents an early event in the apoptosis cascade that precedes the release of pro-apoptotic factors such as cytochrome c and the activation of effector caspases. Based on these findings, we evaluated the effect induced by Pt(IV) derivative of cisplatin containing two axial OA ligands on the Δ*ψ*_m_ in HCT-116 cells by an assay using staining the cells by tetramethylrhodamine, ethyl ester (TMRE) to label active mitochondria. Cells were treated with 1 µM Pt(IV)diOA, its Pt(IV) derivative containing biologically inactive axial acetate ligands [Pt(IV)diAc] or cisplatin for 5 h, stained with TMRE and visualized with confocal laser-scanning microscopy. The representative images are shown in Fig. 5.

Quantitative analysis of TMRE-stained HCT-116 cells revealed a significant reduction (by 26%) in the fraction of cells with an intact Δ*ψ*_m_ relative to untreated control cells (100% intact Δ*ψ*_m_). In contrast, no reduction in the fraction of cells with an intact Δ*ψ*_m_ was observed if the cells were treated with cisplatin or Pt(IV)diAc.

The results of these studies indicate the ability of Pt(IV)diOA to cause a reduction in Δ*ψ*_m_ in HCT-116 tumor cells at a low concentration (in a micromolar range) due to the conjugation of OA to Pt(IV) derivatives of cisplatin. It has been already reported^29–32^ that free OA and some other MCFAs disturb mitochondrial membrane potential. Thus, it is reasonable to suggest that reduction in Δ*ψ*_m_ in HCT-116 tumor cells caused by micromolar concentrations of Pt(IV)diOA can be attributed to the OA ligand(s) released after this Pt(IV) prodrug accumulates in the cell. Hence, these results further support the view that under conditions of our cytotoxicity experiments (Table 2) a significant fraction of the intact molecules of conjugates of OA with Pt(IV) derivative of cisplatin accumulates in cancer cells where it releases OA to reduce their high mitochondrial membrane potential.

### *In vivo* tumor growth inhibition

The syngeneic murine Lewis lung carcinoma (LLC) cells were used in this study to address the *in vivo* cytotoxic activity Pt(IV)diBA, Pt(IV)diHA, Pt(IV)diOA, and Pt(IV)diVPA. LLC is a well-known reproducible syngeneic murine model, highly tumorigenic and is used to model metastasis and to evaluate the efficacy of chemotherapeutic agents *in vivo* ^33^. The advantages of the syngenic mouse model injected with murine cell lines are reproducibility, ability to induce various tumor types, easily and immunocompetence^34^. In addition, because the LLC model can be both syngeneic and orthotopic, tumor microenvironment can be accurately depicted in the animal model.

Xenograft LLC tumors were grown in C57BL mice that were subsequently treated with 1 mgkg^−1^ of either Pt(IV) complex. Pt(IV) prodrugs were administered by oral gavage by taking advantage of their excellent stability, enabling *in vivo* oral formulation making platinum therapy safer lessening side effects and partially overcoming resistance to platinum(II) drugs^2, 35^. The schedule of Pt(IV) prodrug administration *in vivo* was initially determined by assessing the total dose that could be injected without undue toxicity in nontumor-bearing C57BL mice. From day 7 after tumor inoculation, when tumors became visible, tumor-bearing mice received daily doses of the Pt(IV) derivatives (20 mg kg^−1^, by oral gavage). Tumor growth was estimated at day 15, and the results are summarized in Table 4.

**Table 4:** The *in vivo* antitumor activity of Pt(IV) prodrugs and cisplatin evaluated in a model of solid tumor^a^.

	Daily dose (mg kg^−1^)	Average tumor weight (mean ± SD, g)	Inhibition of tumor growth (%)
Control	—	0.542 ± 0.16	—
Pt(IV)diBA	20	0.109 ± 0.05	80
Pt(IV)diHA	20	0.088 ± 0.06	84
Pt(IV)diOA	20	0.025 ± 0.03	95
Pt(IV)diVPA	20	0.174 ± 0.08	68

^a^Lewis lung carcinoma (LLC) was implanted i.m. (2 × 10^6^ cells inoculum) into the right hind leg of 8-week old C57BL mice (24 ± 3 g body weight). Chemotherapy was delayed until the tumor became visible (day 7). Day 7–14: animals received daily orally 20 mg kg^−1^ of Pt(IV) complexes as well as daily i.p. 1.5 mg kg^−1^ of cisplatin. At day 15 animals were sacrificed, legs amputated at the proximal end of the femur, and the inhibition of tumor growth was determined as the difference in weight of the tumor-bearing leg and the healthy leg expressed as % referred to the control animals. The values are the means ± SD of not less than three measurements.

Chemotherapy with platinum complexes showed that all Pt(IV) derivatives were effective in inhibiting tumor growth. Notably, in the case of Pt(IV) complexes containing linear aliphatic chains the therapeutic efficacy increased with the length and lipophilicity of the carboxylate axial ligands. Thus, the highest antitumor activity was observed for Pt(IV)diOA, with an impressive tumor growth inhibition of 95%. Also interestingly, Pt(IV)diOA showed better *in vivo* antitumor activity than the less hydrophobic valproato derivative Pt(IV)diVPA (*vide supra*) also containing C8 chain, but branched.

For the assessment of the adverse side effects, changes in the body weights of tumor-bearing mice were monitored daily over the course of 15 days. The time course of body weight changes (Supplementary Fig. S1) confirmed that treatment with all Pt(IV) complexes did not induce significant adverse effects (body weight loss was lower than 20%), including body weight loss throughout the therapeutic experiment.

It was previously shown for a similar series of dicarboxylato Pt(IV) compounds that organic chains of intermediate length offer the best compromise between lipophilicity and water solubility for oral administration^36^. To better understand the biodistribution properties of the Pt(IV) complexes containing axial aliphatic chains following their administration by oral gavage, tissue samples were collected from LLC-bearing mice treated with a single dose of Pt(IV)diOA and Pt(IV)diVPA and analyzed for platinum content. The details of these experiments and their results are described in the Supplementary material and Supplementary Fig. S2. Platinum concentrations in blood of the mice treated with both Pt(IV)diOA and Pt(IV)diVPA were modest (around 0.5 μg Pt mL^−1^ of whole blood) and slightly higher in the blood of mice treated with Pt(IV)diOA for a longer time of treatment (4 h). Further, the contents of platinum in tissues of the mice treated with both Pt(IV)diOA and Pt(IV)diVPA were similar, and the highest platinum concentrations were found in liver followed by tumor tissue and kidney. In contrast, platinum scarcely accumulated in intestine and lung. Noteworthy, a slightly higher platinum content was found in the tumor mass in the mice treated with Pt(IV)diOA than in those treated with Pt(IV)diVPA. This different platinum content in the tumor mass correlates with the higher *in vivo* antitumor efficacy of Pt(IV)diOA compared to Pt(IV)diVPA (Table 1).

An important factor which may affect the *in vivo* tumor growth inhibition elicited by lipophilic Pt(IV)diVPA or Pt(IV)diOA is their ability to bind lipoproteins and to be transported by lipoproteins in human serum. Actually, protein binding can have important consequences for the *in vivo* pharmacokinetic behavior and may affect bioavailability of these Pt(IV) prodrug. In general, lipophilic drugs are known to be associated to plasma proteins such as albumin^37^ and/or to circulating lipoproteins, which enhances intestinal absorption via lipoprotein encapsulation^38^. In this connection, it is instructive to note that for instance, more than 80% of the immunosuppressive agent cyclosporine A (CyA) is associated with lipoproteins in human serum and this strongly increases its bioavailability^39^, but also deeply limits its uptake in cultured hepatocytes^40^. The trends observed in the results of the *in vivo* antitumor activity experiments, such as the tissue biodistribution (Supplementary Fig. S2A) and blood levels (Supplementary Fig. S2B) suggest that these complexes are efficiently transported *in vivo* and can be sequestrated by plasma proteins differently. On this basis, we carried out *in cellulo* accumulation experiments employing A2780 cells to determine whether the accumulation of Pt(IV)diVPA or Pt(IV)diOA was mainly affected by serum albumin or lipoproteins (for details of these experiments see the Supplementary information and Supplementary Figs S3 and S4).

Our results described in detail in the Supplementary information show that albumin has a poor impact on the accumulation of Pt(IV)diOA or Pt(IV)diVPA. On the other hand, Pt(IV)diOA, in contrast to Pt(IV)diVPA, strongly interacts with lipoproteins, causing a decrease of its short-term cellular accumulation. In fact, only the unbound complex should be susceptible of passive diffusion through the cell membrane^41^. Interestingly, the accumulation of platinum from Pt(IV)diOA or Pt(IV)diVPA after 4 h has been analyzed since this incubation time is close to the half-life of reduction of Pt(IV) derivatives to the active Pt(II) congeners^3^. At least *in vitro*, both Pt(IV)diOA and Pt(IV)diVPA enter cells mainly by passive diffusion, as far as they do not compete with the corresponding free fatty acids and do not interact with copper transporters, as cisplatin does^5^; indeed, Pt(IV)diOA bypasses Ctr-1 linked cisplatin resistance^13^. In this connection, it is instructive to note that for instance, more than 80% of the immunosuppressive agent cyclosporine A (CyA) is associated with lipoproteins in human serum and this strongly increases its bioavailability^39^, but also deeply limits its uptake in cultured hepatocytes^40^. Collectively, these data are consistent with the view and support the hypothesis that also an association of Pt(IV)diOA with lipoproteins may contribute to enhanced bioavailability of this Pt(IV) prodrug in model organisms in comparison with Pt(IV)diVPA.

## Discussion

One approach to improve the toxicity in tumor cells and decrease side effects of platinum anticancer drugs that has received a significant amount of attention is the Pt(IV) prodrug concept^7, 16^. Our study demonstrates that the Pt(IV) derivative of cisplatin, with two axial OA ligands, is a very potent cytotoxic agent against many different cancer cell lines, which overcomes resistance to cisplatin (Table 1) and exhibits promising *in vivo* antitumor activity (Table 4). We show in accord with the previously published reports^10, 13^ that conjugation of the lipophilic medium-chain fatty acid, such as OA, results in markedly enhanced cellular accumulation that correlates with enhanced DNA binding and cytotoxicity.

It has been shown^12^ in the case of other Pt(IV) derivative of cisplatin, having instead of OA axial ligands the branched isomer of OA, i.e. VPA, that its enhanced cytotoxicity is a consequence of several processes. These processes involve besides enhanced cellular accumulation also epigenetic processes consisting in downregulation of HDACs and yet other biochemical processes triggered by VPA released in cells by the reduction of the Pt(IV) prodrug. Moreover, it has also been shown^15^ that non-branched MCFAs may act as HDAC inhibitors. However, our analysis (Fig. 3) confirmed that the efficiency of Pt(IV)diOA or Pt(IV)AcOA to inhibit HDAC activity in tumor cell lines was very small.

Although Pt(IV)diOA was originally designed only to enhance cellular accumulation and DNA platination, once inside the cell cisplatin and OA are released; cisplatin damages DNA and as shown in this report OA simultaneously induces DNA hypermethylation and reduces mitochondrial membrane potential. While consequences of DNA damage by cisplatin for its antitumor effects have been already described in a great detail^42–44^, the significance of the simultaneous effects of DNA hypermethylation and reduction of mitochondrial membrane potential for the mechanism of action of Pt(IV)diOA requires further discussion.

The deregulation of the DNA methylation machinery and aberrant DNA methylation patterns play a critical role in the changes in gene expression involved in cancerogenesis, cancer progression, and metastasis^45^. Widespread global DNA hypomethylation, particularly in DNA repetitive sequences, accompanied by region-specific hypermethylation most often observed in CpG islands in the regions of tumor suppressor genes are associated with the malignant phenotype^46, 47^. The DNA methylation machinery have been considered an attractive target for the anticancer action of new drugs. Two different classes of anticancer drugs are options for targeting DNA methylation: (i) The compounds which reduce the methylation level of CpG islands (for instance inhibitors of DNMTs or compounds promoting the activity of DNA demethylases). (ii) The compounds which globally hypermethylate repeated DNA sequences and in this way inhibit tumor progression caused by global hypomethylation.

The reduction of the methylation level may allow activating tumor suppressor genes, which may suppress tumor progression. This approach has been the focus of many studies but has some weaknesses. For instance, the DNA methylation inhibitors currently in use are not sufficiently specific and, by decreasing methylation, pro-metastasis genes that might increase metastasis could be activated^45^. Moreover, the effectiveness of these inhibitors as anticancer agents is partly owing to cytotoxic effects unrelated to their DNA demethylating activity^47, 48^. DNA methylation inhibitors (e.g. decitabine, 5-azacytidine) are being used as cancer chemotherapeutic agents to treat certain types of cancer, probably those whose origin is mainly associated with hypermethylation of CpG islands in promoters or other tumor suppressor genes^49^. However, these DNA methylation inhibitors could also cause global DNA hypomethylation in other DNA sequences (for instance in DNA repetitive sequences) which are known to promote tumor progression. Conversely, the agents targeting global DNA demethylation or promoting global DNA methylation in certain types of cancer cells may represent potential new epigenetic therapeutics in anticancer chemotherapy. Among the examples of compounds which show anticancer characteristics by methylation of DNA are temozolomide^50, 51^ and S-adenosyl-L-methionine^52, 53^. Thus, we suggest that mechanism of toxicity of Pt(IV)diOA in certain tumor cells may also involve triggering hypermethylation, presumably by stimulating DNMT reactions thereby protecting the genome against global hypomethylation, a hallmark of cancer. Experiments aimed at revealing further details of these epigenetic effects of Pt(IV)diOA are currently in progress.

Collectively, the results of this work support the hypothesis that the mechanism underlying cytotoxicity of Pt(IV) derivatives of cisplatin containing axial OA ligands may involve several processes. Here we show that these processes also comprise: (i) binding of the reduced platinum to DNA much in the same way as cisplatin; (ii) global methylation of the DNA, potentiated by the OA released, upon intracellular reduction; and (iii) the effects of OA released in cancer cells on mitochondria so that Pt(IV)diOA can promote cell death in tumor cells by a mitochondrial-regulated mechanism as well. Therefore, Pt(IV)diOA belongs to the class of “multi-action” antitumor prodrugs triggering several different processes in tumor cells that together orchestrate their death.

## Methods

### Chemicals

#### Synthesis of compounds

K_2_[PtCl_4_] (Johnson Matthey and Co.), octanoyl chloride and 2-propylpentanoyl (valproyl)chloride (Sigma-Aldrich), and all other chemicals were used without further purification. Cisplatin, cis, cis, trans-[Pt(IV)(NH_3_)_2_Cl_2_(BA)_2_] (Pt(IV)diBA), cis, cis, trans-[Pt(IV)(NH_3_)_2_Cl_2_(HA)_2_] (Pt(IV)diHA), cis, cis, trans-[Pt(IV)(NH_3_)_2_Cl_2_(OA)_2_] (Pt(IV)diOA), cis, cis, trans-[Pt(IV)(NH_3_)_2_Cl_2_(VPA)_2_] (Pt(IV)diVPA), cis, cis, trans-[Pt(IV)(NH_3_)_2_Cl_2_AcOA] (Pt(IV)AcOA, and cis, cis, trans-[Pt(IV)(NH_3_)_2_Cl_2_(Ac)_2_] (Pt(IV)diAc) were synthesized according to published procedures^54^. Schematic representation of the platinum compounds used in the present work is shown in Fig. 1.

### Cell lines

The A2780 and A2780cisR human ovarian carcinoma cell lines were grown in RPMI 1640 medium (Biosera) supplemented with gentamycin (50 μg mL^−1^, Serva) and 10% heat-inactivated fetal bovine serum (Biosera). The LNCaP human prostate adenocarcinoma cells were grown in RPMI 1640 medium supplemented with gentamycin (50 μg mL^−1^), 10% heat-inactivated fetal bovine serum and 1% sodium pyruvate (Sigma). The acquired resistance of A2780cisR cells was maintained by supplementing the medium with 1 μM cisplatin every second passage. The MCF7 (human breast cancer cells), MRC5 pd30 (human fetal lung fibroblasts) and CHO-K1 (Chinese hamster ovary cells) were grown in DMEM medium (high glucose, 4.5 g L^−1^, Biosera) supplemented with gentamycin (50 μg mL^−1^, Serva) and 10% heat-inactivated fetal bovine serum (Biosera). The HCT-116 human colorectal carcinoma cells were cultivated in McCoy´s 5a medium (Sigma) supplemented with gentamycin (50 μg mL^−1^), 10% heat-inactivated fetal bovine serum. The HSF human primary skin fibroblasts were grown in DMEM supplemented with streptomycin (100 μg mL^−1^), penicillin (100 U mL^−1^), and 10% heat inactivated FBS. The A2780, A2780cisR, MCF7, CHO-K1, HCT-116 cells were kindly supplied by prof. Keppler (Vienna, Austria). The LNCaP and MRC-5 pd30 were from European collection of cell cultures (ECACC). HSF cells (primary cell culture) was a kind gift from Professor T. Adam (Department of Clinical Chemistry, Palacky University and Hospital, Olomouc, Czech Republic). The cells were cultured in a humidified incubator at 37 °C in a 5% CO_2_ atmosphere and subcultured 2–3 times a week with an appropriate plating density.

### Cytotoxicity (*in cellulo* experiments)

Cell death was evaluated using a system based on the tetrazolium compound MTT [3-(4,5-dimethyl-2-thiazolyl)-2,5-diphenyl-2H-tetrazolium bromide]. The cells were seeded in 96-well tissue culture plates at a density of 10^4^ A2780cisR, LNCaP, HSF cells/well, 3 × 10^3^ CHO-K1 or 4 × 10^3^ MCF7, HCT-116 and MRC5 pd30 cells/well in 100 μL of medium. After overnight incubation (16 h), the cells were treated with the compounds in a final volume of 200 μL/well. After additional 72 h, 10 μL of a freshly diluted MTT solution (2.5 mg mL^−1^) was added to each well, and the plate was incubated at 37 °C in a humidified 5% CO_2_ atmosphere for 4 h. At the end of the incubation period, the medium was removed, and the formazan product was dissolved in 100 μL of DMSO. The cell viability was evaluated by measurement of the absorbance at 570 nm, using an Absorbance Reader SUNRISE TECAN SCHOELLER. IC_50_ values were calculated from curves constructed by plotting cell survival (%) versus drug concentration (μM). All experiments were made in triplicate. The reading values were converted to the percentage of control (% cell survival). Cytotoxic effects were expressed as IC_50_.

### Accumulation of platinum in tumor cells treated with Pt(IV) complexes and cisplatin (*in cellulo* experiments)

Cellular accumulation of platinum from complexes Pt(IV)diOA, Pt(IV)AcOA, Pt(IV)diAc and cisplatin was measured in A2780 and HCT-116 cells. The cells were seeded in 150 cm^2^ culture flasks at a density 1 × 10^7^ cells/flask (67 000 cells/cm^2^). After 48-h incubation, the cells were treated with the compounds (0.35 μM) for 24 h. The attached cells were washed twice with PBS (4 °C). Seventy-five percent of cells were used for analysis of platinum bound to DNA, remaining 25% were used for estimations of cellular accumulation of platinum. Cell pellets were digested by a high-pressure microwave digestion system (MARS5, CEM) with HCl to give a fully homogenized solution, and final platinum content was determined by ICP-MS. The analysis with the aid of ICP-MS was performed using Agilent 7500 (Agilent, Japan) or Thermo Optek X Series.

### Platinum-DNA binding in tumor cells treated with Pt(IV) complexes and cisplatin (*in cellulo* experiments)

Cellular uptake of complexes Pt(IV)diOA, Pt(IV)AcOA, Pt(IV)diAc and cisplatin was measured in A2780 cells. The cells were seeded in 150 cm^2^ culture flasks at a density 1 × 10^7^ cells/flask (67 000 cells/cm^2^). After 48-h incubation, the cells were treated with the compounds (0.35 μM) for 24 h. The attached cells were washed twice with PBS (4 °C). As mentioned in the preceding paragraph, 75% of cells were used for analysis of platinum bound to DNA, remaining 25% were used for estimations of cellular accumulation of platinum. Cells were then lysed in DNAzol (DNAzol® genomic DNA isolation reagent, MRC) supplemented with RNase A (100 μg mL^−1^). The genomic DNA was precipitated from the lysate with ethanol, dried and resuspended in water. The DNA content in each sample was determined by UV spectrophotometry. The DNA samples were digested in the presence of hydrochloric acid (11 M) using high-pressure microwave mineralization system (MARS5, CEM). The platinum content was determined by ICP-MS. Experiments were performed in triplicate and the values are the means ± SD.

### Measurement of lipophilicity

To determine the partition coefficient (P) the shake flask method was used. Octanol-saturated water (OSW) and water-saturated octanol (WSO) were prepared using analytical grade octanol and 0.2 M aqueous NaCl solution (to suppress hydrolysis of the chlorido complexes). Compounds were first dissolved in DMF (compounds Pt(IV)diOA, Pt(IV)AcOA) or water (Pt(IV)diAc and cisplatin). Mixing was done by vortexing for 30 min at room temperature to establish the partition equilibrium. To separate the phases, centrifugation was done at 3000 g for 5 min. The aqueous layer was carefully separated from the octanol layer for platinum analysis. Platinum was quantified from aliquots taken from the octanol-saturated aqueous samples before and after partition by ICP-MS. Partition coefficients of Pt complexes were calculated using the equation log P = log ([Pt]WSO/[Pt]OSW).

### Cellular distribution, preparation of cytosolic and nuclear extracts

A2780 cells were seeded in 100 mm tissue culture dishes (3 × 10^6^ cells) and grown for 48 h to 80–90% confluence. Cells were harvested after 24 h of treatment. Concretely, the dishes were washed two times with ice-cold PBS and the cells scraped into tubes and centrifuged for 10 min at 0 °C (200 g). The pellets were resuspended in the lysis buffer [Tris·HCl (10 mM, pH 8.0), KCl (60 mM), EDTA (1.2 mM), DTT (1 mM), phenylmethanesulfonyl fluoride (PMSF, 0.1 mM), NP-40 (0.04%)] for 10 min on ice and centrifuged for 4 min at 0 °C (800 g). Cytosolic fraction was separated for platinum ICP-MS analysis, and protein concentration was determined by Bradford assay. The pellets were rinsed with the above lysis buffer without PMSF and NP-40 and centrifuged for another 4 min at 0 °C (300 g). The pellets were resuspended in 50 µL nuclear extraction buffer [Tris·HCl (20 mM, pH 8.0), NaCl (420 mM), MgCl_2_ (0.7 mM), EDTA (0.25 mM), glycerol (25%)] and incubated for 30 min at 4 °C, continuously mixed on end-over-end roller, and then centrifuged for 15 min at 0 °C (13000 g). Protein concentration in nuclear extracts was determined by the Bradford assay, and stored at −80 °C until analysis.

### Analysis of the epigenetic changes after the treatment of HCT-116 cells with Pt(IV)diOA, Pt(IV)AcOA and cisplatin

#### Activity of HDACs in HCT-116 cells

The activity of HDAC enzymes was analyzed in HCT-116 cells treated with compounds tested in the present work (1 µM). The cells were incubated for 0, 1, 3 and 5 h with Pt(IV) conjugates and for comparative purposes also with free OA (1 µm or 1 mM) and the inhibitor of HDACs trichostatin A (TSA). The activity of HDAC was analyzed using the HDAC fluorimetric cellular activity assay kit (Enzo Life Sciences; Farmingdale, NY) in the whole cells according to manufacturer’s instructions. Briefly, the HCT-116 cells were seeded and incubated for 24 h to gain desired 80% confluence in the culture media without phenol red. Incubations with the compounds tested in the present work and the HDAC substrate were begun in the reverse staggered fashion and incubated for indicated times at 37 °C. Samples were treated in octuplicate, and each experiment was repeated in triplicate. HDAC activity was terminated by addition of 2 µM of TSA and subsequent incubation for 15 min at 37 °C with the developer to generate a fluorescent signal from the deacetylated substrate. The plates were scanned on a fluorescence reader Infinite 200 (TECAN) (360 nm excitation, 460 nm emission).

#### DNA methyltransferase (DNMT) activity assay

Human colon carcinoma cells (HCT-116) were seeded in 100 mm culture dishes at the density 1.5 × 10^6^ cells/dish. Cells were harvested after 24 h of incubation with the compounds tested in the present work (1 µM). Nuclear extracts from the pellets were separated using EpiQuik Nuclear extraction kit I (Epigentek, NY) according to manufacturer’s instructions. Quantification of nuclear proteins was analyzed with the standard Bradford assay. The activity of DNMT enzymes was analyzed using the EpiQuik DNMT activity/inhibition assay Ultra kit (Epigentek, NY), according to the manufacturer´s protocol. Briefly, 10 µg of total nuclear protein was analyzed for its DNMT activity. The reaction time with a universal DNMT substrate stably coated onto microplate wells was 120 min at 37 °C. The final signal at the end of the ELISA reaction was detected at 450 nm (reference 620 nm). DNMT activity was normalized to controls and expressed in the percent or absolute values as OD h^−1^ mg^−1^, calculated as described in the manufacturer’s manual.

#### DNA methylation analysis

Human colon carcinoma cells (HCT-116) were seeded at the density 1.5 × 10^6^ cells/dish. Cells were harvested after 24 or 72 h of incubation with the compounds tested in the present work (0.5 or 1 µM) (incubation with cisplatin was also performed at its concentration of 30 µM and with free OA was also performed at its concentration of 1 mM). The attached cells were washed twice with PBS (4 °C) and then lysed in DNAzol (DNAzol® genomic DNA isolation reagent, MRC) supplemented with RNAse A (100 μg mL^−1^). The genomic DNA was precipitated from the lysate with ethanol, dried and resuspended in water. The DNA content in each sample was determined by UV spectrophotometry. DNA (200 ng) from the samples was loaded to the Imprint methylated DNA quantification kit (Sigma-Aldrich, USA). The level of methylated DNA was proportional to the absorbance measured at 450 nm.

### Analysis of mitochondrial function

#### Cellular treatment, fluorescence staining

HCT-116 cells were seeded on glass bottom dishes (P50G-0-30-F, MatTek Co., Ashland, USA) at the density 3 × 10^5^ cells per dish, cultured in RPMI-1640 medium (without phenol red) and incubated at 37 °C in a humidified 5% CO_2_ atmosphere overnight. The cells were subsequently treated with the compounds (1 μM) for 5 h. After this period, cells were washed twice with PBS and resuspended in staining medium (without Pt compound), supplemented with Hoechst 33258 and tetramethylrhodamine, ethyl ester (TMRE) from multiparametric apoptosis assay kit (Cayman Chemical, Michigan, USA) according to the manufacturer’s protocol. After 20 min of staining, the staining medium was replaced by a fresh medium (without staining dye and platinum compound).

#### Confocal microscopy

Images of living cells were acquired with a Leica TSC SP-5X laser scanning confocal microscope (Leica Microsystems, Mannheim, Germany) using an HCX PL APO lambda blue 63.0 × 1.20 water UV objective lens corrected with an appropriate beam path (resolution 1024 × 1024, frequency 100 Hz). Excitation/emission wavelengths: Hoechst 33258 (355/465 nm), TMRE (560/595 nm). UV laser for excitation of Hoechst 33258 was used at the power of 6.4 mW, TMRE was excited by supercontinuum white light laser at the power of 5.1 mW. Emission PMT detectors were set to the voltage of 900 V (Hoechst) and emission hybrid detectors were set to the gain of 300%. For better morphological and structural resolution of the cell nucleus, the pinhole size was reduced to 70 μm corresponding to 0.63 Airy and the theoretical image voxel width and/or height was 108 nm. Quantification of the mean signal intensity associated with single cells was performed using software ImageJ v1.47, U.S. National Institutes of Health, Bethesda, Maryland, USA.

### Experiments with animals

All studies involving animal testing were carried out in accordance with the ethical guidelines for animal research adopted by the University of Padua, acknowledging the Italian regulation (D.L.G.S. 116/92) and European Directive 86/609/EEC as to the animal welfare and protection and the related codes of practice. The experimental protocol was approved by the Italian Health Department according to the art. 7 of above mentioned D.L.G.S. 116/92. The mice were purchased from Charles River, Italy, housed in steel cages under controlled environmental conditions (constant temperature, humidity, and 12 h dark/light cycle), and alimented with commercial standard feed and tap water ad libitum.

#### *In vivo* antitumor activity in Lewis Lung Carcinoma

The LLC cell line was purchased from ECACC, United Kingdom. The LLC cell line was maintained in DMEM (Euroclone) supplemented with 10% heat-inactivated fetal bovine serum (FBS; Euroclone), 10 mM L-glutamine, 100 U mL^−1^ penicillin, and 100 μg mL^−1^ streptomycin in a 5% CO_2_ air incubator at 37 °C. The LLC was implanted intramuscularly (im) as a 2 × 10^6^ cell inoculum into the right hind leg of 8-week old male and female C57BL mice (24 ± 3 g body weight). After 24 h from tumor implantation, mice were randomly divided into five groups (8 animals per group, 10 controls) and treated orally with a single daily dose of butanoate, hexanoate, octanoate and valproate Pt(IV) complexes (20 mg kg^−1^ dissolved in a vehicle solution composed of 20% Cremophor EL (v/v), 20% PEG400 (v/v) and 60% saline solution (v/v)) or with a daily ip dose of cisplatin (1.5 mg kg^−1^ in 0.9% NaCl solution) from day 7 after tumor inoculation (visible tumor). At day 15, animals were sacrificed, the legs were amputated at the proximal end of the femur, and the inhibition of tumor growth was determined according to the difference in weight of the tumor-bearing leg, and the healthy leg of the animals expressed as a percentage referred to the control animals. Body weight was measured every two days and was taken as a parameter for systemic toxicity. All the values are the means ± SD of not less than three measurements.

### Statistical analysis

All the values are the means ± SD of not less than three independent experiments. Multiple comparisons were made by ANOVA followed by the Tukey–Kramer multiple comparison tests (***P < 0.001, **P < 0.01; *P < 0.05) using GraphPad Software.

## Electronic supplementary material

Supplementary Material
