# Supplementary material for: Epigenetic and antitumor effects of platinum(IV)-octanoato conjugates
Source: Sci Rep. 2017 Jun 16;7:3751. doi: 10.1038/s41598-017-03864-w (PMC5473904; doi:10.1038/s41598-017-03864-w)
Supplement: Supplementary file 1 — Supplementary Material [file 41598_2017_3864_MOESM1_ESM.pdf]

Supplementary Material

Epigenetic and antitumor effects of platinum(IV)-octanoato conjugates

Vojtech Novohradsky[1], Ilaria Zanellato[2], Cristina Marzano[3], Jitka Pracharova[4], Jana Kasparkova[1], Dan Gibson[5], Valentina Gandin[3], Domenico Osella*[2] & Viktor Brabec*[1]

[1] Institute of Biophysics, Academy of Sciences of the Czech Republic, v.v.i., Kralovopolska 135, CZ-61265 Brno, Czech Republic
[2] Dipartimento di Scienze e Innovazione Tecnologica, Universita del Piemonte Orientale "A. Avogadro" Viale T. Michel 11, 15121, Alessandria, Italy
[3]Dipartimento di Scienze del Farmaco, Universita di Padova, Via Marzolo 5, 35131 Padova, Italy
[4] Department of Biophysics, Centre of the Region Hana for Biotechnological Agricultural Research, Faculty of Science, Palacky University, 17. listopadu 12, CZ-77146 Olomouc, Czech Republic
[5] Institute for Drug Research, School of Pharmacy, the Hebrew University, Jerusalem 91120, Israel

Table of Contents

RESULTS

The body weight changes of LLC-bearing C57BL mice treated with vehicle or platinum compounds (Figure S1) S2

Biodistribution after oral administration (Figure S2) S2

Interactions with serum proteins (Figures S3 and S4) S4

METHODS

Biodistribution after oral administration S5

Accumulation of platinum in tumor cells treated with Pt(IV) complexes and cisplatin (in cellulo experiments) in the absence of serum S5

REFERENCES S6

RESULTS

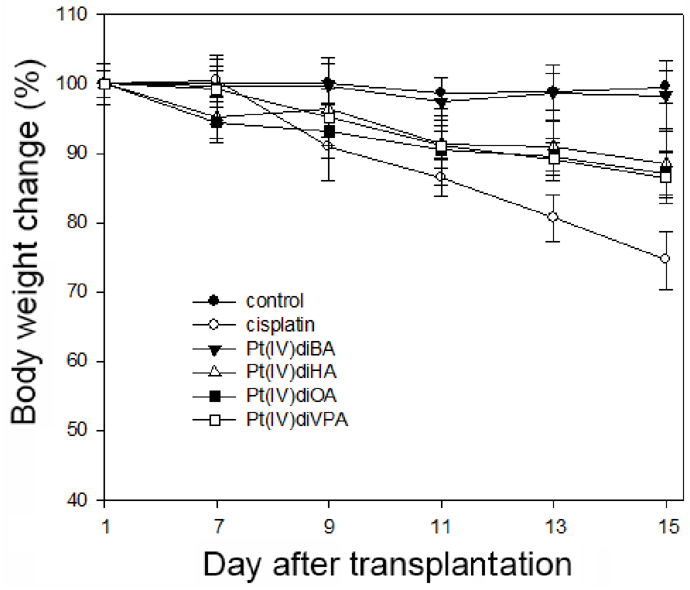

Supplementary Figure S1: Body weight changes
The body weight changes of LLC-bearing C57BL mice treated with vehicle or platinum compounds. Body weight was measured at day 1 and every two days from day 7 and was taken as a parameter of systemic toxicity. The error bars indicate the SD of not less than three measurements.

Biodistribution after oral administration

For Pt(IV) complexes having axial carboxylato ligands, the lipophilicity increases as the number of carbon atoms in the chain increases. This lipophilicity increase offers a beneficial effect on the cellular accumulation and *in vitro* cytotoxic potency up to a point where the drop in water solubility impedes the administration of the drugs (around C8-C10 carbon chains)[1,2]. Moreover, excessive lipophilicity can hinder transport of such Pt(IV) derivatives *in vivo* across intestinal epithelial cells making them more vulnerable to hepatic metabolism.

It was previously shown for a similar series of dicarboxylato Pt(IV) compounds (except the VPA-derivative) that organic chains of intermediate length offer the best compromise between lipophilicity and water solubility for oral administration[3]. To better understand the biodistribution properties of the Pt(IV) complexes, following administered by oral gavage in a single dose (20 mg kg^{-1}), tissue samples collected from LLC-bearing mice were analyzed by ICP-MS. After 1 and 4 h, blood samples were collected and subsequently animals were sacrificed, and tissue samples were collected. The platinum levels in tumor, kidney, liver, intestine, lung and whole blood aliquots are shown in Fig. S2, panels A and B. It is important to recall that using this protocol it is possible to estimate only the overall Pt content and not its oxidation state or speciation [i.e. Pt(IV) metabolites or Pt(II) metabolites produced by reduction and further interactions]. Fig. S2B clearly shows that both Pt(IV)diOA and Pt(IV)diVPA exhibit, as expected, modest Pt concentrations in blood (around 0.5 µg Pt mL^{-1} of whole blood), where at 4 h that of Pt(IV)diOA is somewhat higher than that of Pt(IV)diVPA.

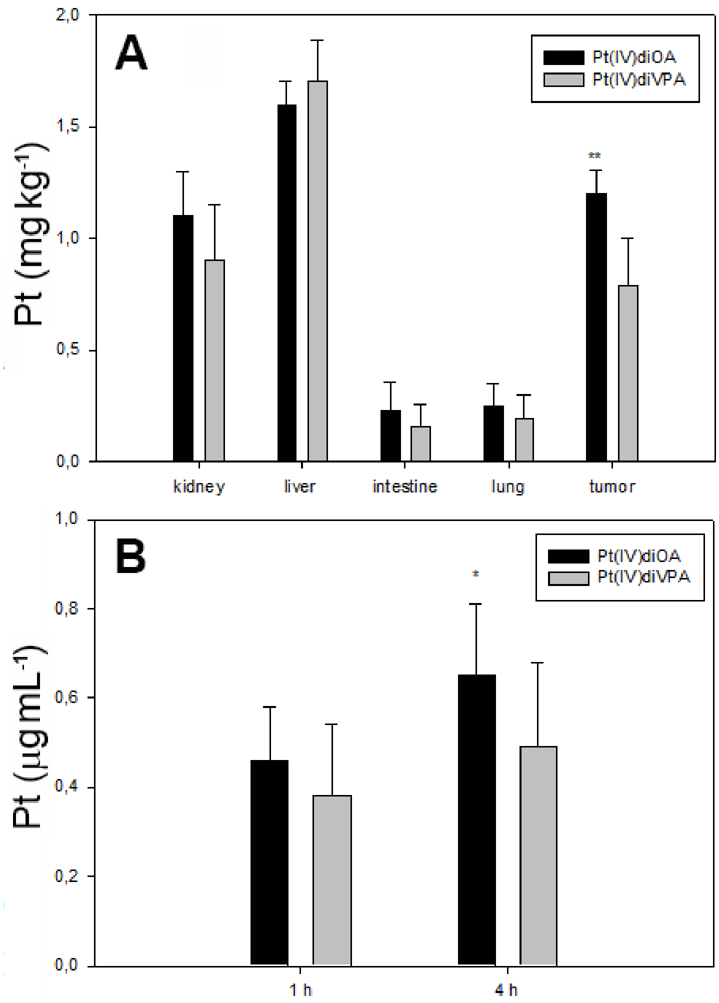

Supplementary Figure S2: Platinum levels determined in organs of mice treated with Pt(IV) prodrugs

Total platinum levels determined in organs of mice treated with Pt(IV)diVPA or Pt(IV)diOA (A) or blood samples (B) after single dose oral application (20 mg kg^{-1}). The error bars indicate the SD of not less than three measurements. Multiple comparisons were made by the Tukey− Kramer test (**, $p < 0.01$; * $p < 0.05$).

The different hematic levels of platinum from the Pt(IV)diOA and Pt(IV)diVPA complexes can be attributed to the different absorption and disposition of the bound fatty acids. Both complexes contain MCFAs: this class of fatty acids is used to increase the delivery of drugs with low oral availability[4]; moreover, water-in-oil (w/o) microemulsions containing C8-12 acids improved intestinal absorption and bioavailability[5]. In particular, OA is immediately available after gastric absorption for acylation of ghrelin, the peptide hormone with an orexigenic effect[6]. Intriguingly, the presence of the OA-acyl group is necessary for ghrelin interaction with lipoproteins[7].

Tissue platinum contents (expressed as mg·kg^{-1}) were similar for both Pt(IV)diOA and Pt(IV)diVPA compounds (the percentage of the administered drug reaching the tumor mass was about 18% and 10%, respectively) and the highest platinum concentrations were found in liver followed by tumor tissue and kidney. On the contrary, complexes were scarcely accumulated in intestine and lung. Noteworthy, in Pt(IV)diOA treated LLC mice a slightly higher platinum content was recorded in the tumor mass. These data concerning tumor

targeting ability well correlate with the higher *in vivo* antitumor potential of Pt(IV)diOA compared to Pt(IV)diVPA.

Interactions with serum proteins

The trends observed in the results of the *in vivo* antitumor activity experiments, such as the tissue biodistribution (Fig. S2A), blood levels (Fig. S2B), and cellular accumulation (Table 2) of Pt(IV) complexes could be also related to their sequestration by plasma proteins, mainly by human serum albumin[8]. Thus, the accumulation experiments *in cellulo* were carried out employing A2780 cells without serum (and therefore without BSA) in the culture medium. As shown in Fig. S3, when passing from the treatment in complete medium (+FBS) to the treatment in serum-free medium (-FBS), no significant change was observed for Pt(IV)diVPA ($p>0.01$, two-sample t-test), whereas a significant 3-fold increase was observed for Pt(IV)diOA.

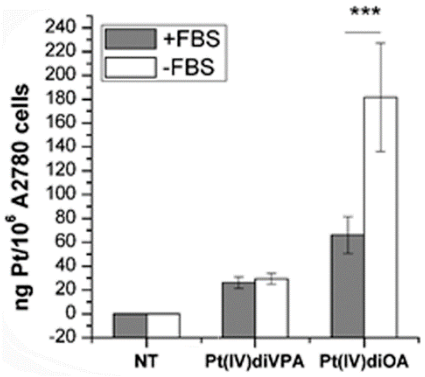

Supplementary Figure S3: Pt accumulation in A2780 cells treated with Pt(IV) prodrugs Pt accumulation in A2780 cells of control (NT), Pt(IV)diVPA and Pt(IV)diOA after 4h of treatment in complete medium (+FBS) and in serum-free medium (- FBS). Data are means of at least three replicates and were tested using the two sample t-test (***$p<0.001$).

Surprisingly, the presence of serum affects only the accumulation of Pt(IV)diOA. The medium supplemented with 10% FBS contains 30 μM BSA, which is known to transport octanoate[9,10], and 300 μg mL^{-1} cholesterol in the most lipophilic component, i.e. lipoproteins[11], which are known to transport lipophilic drugs[12,13]. To verify whether the accumulation of Pt(IV)diOA could be mainly affected by BSA or lipoproteins, the experiments were repeated in serum-free medium supplemented with BSA (-FBS, + BSA) or LPC, lipoprotein-rich cholesterol (-FBS + LPC), respectively (see experimental part). As shown in Fig. S4, while the BSA addition gave a negligible effect ($p>0.01$, two-sample t-test), the LPC supplementation decreased Pt accumulation.

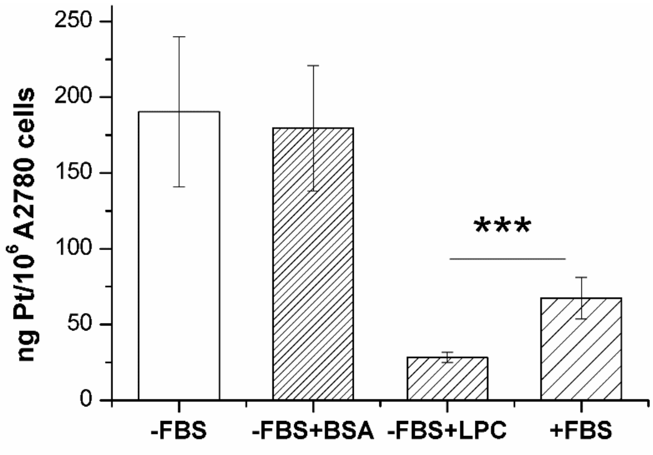

Supplementary Figure S4: Pt accumulation in A2780 cells treated with Pt(IV)diOA prodrug

Pt accumulation in A2780 cells after 4 h of treatment with Pt(IV)diOA, in serum-free medium (control, -FBS), in serum-free medium supplemented with 30 μM BSA (-FBS, + BSA), with lipoproteins (-FBS + LPC), and in complete medium (+FBS). Data are means of at least three replicates and were tested using the two sample t-test (***p<0.001).

METHODS

Biodistribution after oral administration. For *in vivo* kinetic experiments, 8-10 week old C57BL mice (24 ± 3 g body weight) received the drugs as an oral gavage single dose of butanoato, hexanoato, octanoato and valproate Pt(IV) complexes (20 mg kg^{-1}, dissolved in a vehicle solution composed of 20% Cremophor EL (v/v), 20% PEG400 (v/v) and 60% saline solution (v/v)). At each time point (1 and 4 h, respectively), animals were anesthetized, and blood was collected by a vain tail puncture. In addition, samples of organs (kidney, liver, lung and intestine) and tumor were collected at 4 h and stored at −20 °C. Blood, organ samples and tumor were subjected to quantitative determination of platinum content. The samples were treated with highly pure nitric acid ([Pt] ≤ 0.01 μg kg^{-1} TraceSELECT Ultra, Sigma Chemical Co.) and transferred into a microwave Teflon vessel. Subsequently, samples were submitted to the standard procedure using a speed microwave. After cooling, each mineralized sample was analyzed for platinum by ICP-MS (ThermoOptek X Series 2). Instrumental settings were optimized to achieve maximum sensitivity for platinum. For quantitative determination, the most abundant isotopes of platinum and indium (used as internal standard) were measured at m/z 195 and 115, respectively.

Accumulation of platinum in tumor cells treated with Pt(IV) complexes and cisplatin (*in cellulo experiments*) in the absence of serum. A2780 cells were seeded in 10 mm Petri dishes and treated with the platinum complexes (10 μM) for 4 h in complete medium, i.e. + 10 % FBS (HyClone, GE Healthcare). At time zero, 100 μL of medium was taken out from each sample to check the extracellular Pt concentration. At the end of the exposure, cells were washed three times with phosphate-buffered saline (PBS), detached from the Petri dishes using 0.05% Trypsin 1X + 2% EDTA (HyClone, GE Healthcare) and harvested in fresh complete medium. An automatic cell counting device (Countess®, Life Technologies) was used to measure the number and the mean diameter from every cell count. To unravel the possible factors affecting the Pt(IV) bioavailability, a parallel of cellular accumulation experiment was performed in the absence of serum (-FBS). Moreover, taking into account the amount of bovine serum albumin (BSA) and cholesterol in lipoproteins that 10% FBS brings

to the complete medium, the medium deprived of FBS was supplemented with an equivalent amount of BSA(- FBS + BSA) or with an equivalent amount of cholesterol in lipoproteins (Lipoprotein-rich cholesterol, LPC, MP Biomedicals) (-FBS + LPC). For the cellular Pt accumulation analysis, the cells were transferred into a borosilicate glass tube and centrifuged at 1100 rpm for 5 min at room temperature. The supernatant was carefully removed by aspiration, while about 200 μL of the supernatant was left to limit the cellular loss. Cellular pellets were stored at −80 °C until mineralization.

The level of Pt found in cells after drug treatment and normalized upon the cell number (cellular Pt accumulation) was expressed as ng Pt per 10^6 cells. Mineralization was performed by the addition of 70% w/w HNO_3 to each sample (after defrosting), followed by incubation for 1 h at 60 °C in an ultrasonic bath. Before the ICP-MS measurement, the HNO_3 was diluted to a final 1% concentration.

References

1. Varbanov, H. P. *et al.* Theoretical investigations and density functional theory based quantitative structure-activity relationships model for novel cytotoxic platinum(IV) complexes. *J. Med. Chem.* **56**, 330-344 (2012).
2. Gramatica, P. *et al.* Antiproliferative Pt(IV) complexes: synthesis, biological activity, and quantitative structure-activity relationship modeling. *J. Biol. Inorg. Chem.* **15**, 1157-1169 (2010).
3. Zanellato, I. *et al.* Biological activity of a series of cisplatin-based aliphatic bis(carboxylato) Pt(IV) prodrugs: How long the organic chain should be? *J. Inorg. Biochem.* **140**, 219-227 (2014).
4. Lindmark, T., Kimura, Y. & Artursson, P. Absorption enhancement through intracellular regulation of tight junction permeability by medium chain fatty acids in Caco-2 cells. *J. Pharmacol. Exp. Ther.* **284**, 362-369 (1998).
5. Constantinides, P. P. *et al.* Water-in-oil microemulsions containing medium-chain fatty acids salts: Formulation and intestinal absorption enhancement evaluation. *Pharmaceut. Res.* **13**, 210-215 (1996).
6. Lemarie, F., Beauchamp, E., Legrand, P. & Rioux, V. Revisiting the metabolism and physiological functions of caprylic acid (C8:0) with special focus on ghrelin octanoylation. *Biochimie* **120**, 40-48 (2016).
7. De Vriese, C., Hacquebard, M., Gregoire, F., Carpentier, Y. & Delporte, C. Ghrelin interacts with human plasma lipoproteins. *Endocrinology* **148**, 2355-2362 (2007).
8. Zheng, Y. R. *et al.* Pt(IV) prodrugs designed to bind non-covalently to human serum albumin for drug delivery. *J. Am. Chem. Soc.* **136**, 8790-8798 (2014).
9. Kragh-Hansen, U. Structure and ligand binding properties of human serum albumin. *Dan. Med. Bull.* **37**, 57-84 (1990).
10. Chen, Y. M. & Guo, L. H. Combined fluorescence and electrochemical investigation on the binding interaction between organic acid and human serum albumin. *J. Environ. Sci.* **21**, 373-379 (2009).
11. Rudman, D., Hollins, B., Bixler, T. J. & Mosteller, R. C. Transport of drugs, hprmones and fatty-acids in lipemic serum. *J. Pharmacol. Exp. Ther.* **180**, 797-810 (1972).
12. Wasan, K. M., Brocks, D. R., Lee, S. D., Sachs-Barrable, K. & Thornton, S. J. Impact of lipoproteins on the biological activity and disposition of hydrophobic drugs: implications for drug discovery. *Nature Rev. Drug Discov.* **7**, 84-99 (2008).
13. Patel, J. P. & Brocks, D. R. The effect of oral lipids and circulating lipoproteins on the metabolism of drugs. *Exp. Opin. Drug Metabol. Toxicol.* **5**, 1385-1398 (2009).
